# Changes in food allergy and sensitisation over a decade: two birth cohorts from the same geographical area

**DOI:** 10.1186/2045-7022-3-S3-O20

**Published:** 2013-07-25

**Authors:** VK Patil, C Venter, J Grundy, R Kurukulaaratchy, T Dean, SH Arshad

**Affiliations:** 1 David Hide Asthma & Allergy Research Centre, Newport, UK; 2 CES, University of Southampton, Southampton, UK; 3 SHSSW, University of Portsmouth, Portsmouth, UK

## Background

Prevalence of food allergy is considered to be increasing with some even suggesting a food allergy epidemic. We aimed to look at the changes in reported symptoms of food allergy and food allergen sensitisation over a 10 year period in the same geographical area. We compared the prevalence of reported food allergy and food allergen sensitisation in 9-10 year old children between the Isle of Wight (IoW) birth cohort and Food Allergy and Intolerance Research (FAIR) cohort.

## Methods

Subjects from the 1989 IOW birth cohort (n=1456) were followed up at 1, 2, 4, 10 and 18 years; the 10 year follow up was completed in 1999-2001. The FAIR cohort was established in 2001 (n=988) and children were followed up at 1, 3 and 9 years; the 9 year follow up was completed in 2010-2011. Reported symptoms were based on the questionnaire data and sensitisation to food allergens was defined as ≥3mm response on skin prick test (SPT).

## Results

Reported food allergy information was available for 1368 and 827 children and SPT was available for 1034 and 588 children in the IoW and FAIR cohort respectively. Reported allergy to Milk (1.2% to 2.1%), Egg (0.7% to 1.6%), Peanut (1.1% to 1.7%) showed an increase but not statistically significant. Reported allergy to Fish (0.4% to 0.1%) decreased but this was not significant. Statistically significant increase was seen for wheat (0.3% to 1.9%, p<0.001). Allergic sensitisation to Milk (0.3% to 0.3%), Egg 0.4% to 0.3%), Peanut (1.7% to 2.4%) and Fish (0.8% to 0.2%) did not show significant change.

## Conclusion

There was no significant increase in reported food allergy or sensitisation over a decade for milk, egg, fish and peanut. There is an increase in reported allergy to wheat.

## Disclosure of interest

None declared.

